# Modeling and Evaluation of Customizable Immobilization Masks for Precision Radiotherapy

**DOI:** 10.3390/polym18020287

**Published:** 2026-01-21

**Authors:** Diana Adlienė, Antonio Jreije, Paulius Griškevičius, Neringa Keršienė, Rūta Nedzinskienė

**Affiliations:** 1 Department of Physics, Kaunas University of Technology, Studentu Str. 50, 51368 Kaunas, Lithuania; 2 Department of Mechanical Engineering, Kaunas University of Technology, Studentu Str. 56, 51424 Kaunas, Lithuania; 3 School of Economics and Business, Kaunas University of Technology, Gedimino Str. 50, 44029 Kaunas, Lithuania

**Keywords:** radiotherapy, head and neck immobilization mask, metal-reinforced thermoplastic, additive manufacturing, modeling

## Abstract

Accurate immobilization is critical in head and neck (H&N) radiotherapy to ensure precise dose delivery while minimizing irradiation of surrounding healthy tissues. However, conventional thermoplastic masks cannot secure 100% replicas of the patient’s surface and are often limited by mechanical weakness, patient discomfort, and workflow inefficiencies. Recently, the best replicas of the patient’s face have been obtained by exploring personal CT or MRI scans of patients that are used for manufacturing of immobilization masks. This study aimed to design and evaluate customizable immobilization masks using acrylonitrile butadiene styrene (ABS)-based composites reinforced with bismuth oxide (Bi_2_O_3_) and to compare their mechanical performance against commercial thermoplastic masks. ABS and ABS/Bi_2_O_3_ composite filaments (5, 10, and 20 wt%) were fabricated and characterized by tensile testing. A patient-specific virtual mask was modeled and subjected to finite element analysis (FEA) under clinically relevant loading scenarios, including neck flexion and lateral bending. Results were benchmarked against two commercial thermoplastic masks. ABS and ABS-based composites exhibited significantly higher stiffness (1.7–2.5 GPa) and yield strength (20–25 MPa) compared to commercial thermoplastics (0.25–0.3 GPa, ~7 MPa; *p* < 0.001). FEA simulations revealed markedly reduced displacement in ABS masks (1–5 mm at 2 mm thickness; <1 mm at 4 mm thickness) relative to commercial masks, which exceeded 20 mm under lateral load. Hybrid configurations with reinforced edges further optimized rigidity while limiting material usage. Customized ABS-based immobilization masks outperform conventional thermoplastics in mechanical stability and displacement control, with the potential to reduce planning margins and improve patient comfort. In addition, ABS-based masks can be recycled, and Bi_2_O_3_-filled composites can be reused for printing new immobilization masks, thus contributing to a reduced amount of plastic waste. These findings support their promise as next-generation immobilization devices for precision radiotherapy, warranting further clinical validation, workflow integration and sustainable implementation within a circular economy.

## 1. Introduction

Radiotherapy is a cornerstone in the treatment of head and neck (H&N) cancers. Its clinical effectiveness relies heavily on the ability to deliver high radiation doses to the tumor with millimetric precision while sparing adjacent healthy tissues. The anatomical complexity of the H&N region, with critical organs at risk such as the spinal cord, brainstem, optic nerves, and salivary glands, makes precise targeting essential. Even minor setup errors, as small as 1 mm, have been shown to reduce tumor coverage and compromise treatment conformity [1,2]. To mitigate these risks, reproducible patient immobilization across treatment fractions is vital.

Thermoplastic masks are the standard immobilization device used in H&N radiotherapy. These masks are molded from a perforated thermoplastic sheet that softens when heated and conforms to the patient’s facial contours [3]. Once cooled, the mask hardens into a rigid shell that securely attaches to a baseplate, enabling consistent patient positioning. Depending on the type of thermoplastic material used, residual setup errors can typically be limited to within 2–5 mm [1]. To account for these uncertainties, a planning margin of approximately 3–4 mm is often added around the clinical target volume [1]. This immobilization approach has become an integral part of conformal and intensity-modulated radiotherapy techniques.

Despite their widespread use, traditional thermoplastic masks present several challenges related to patient comfort, procedural complexity, and adaptability. The fabrication process requires the patient to remain motionless while a warm, damp sheet of thermoplastic is applied and molded over the face, an experience that can be uncomfortable and, for many, claustrophobic [4]. The snug enclosure, often extending over the eyes, nose, and mouth, can trigger significant anxiety [2]. In fact, approximately 25–50% of H&N radiotherapy patients experience distress linked to mask use, with studies by Nixon et al. (2018) and Elsner et al. (2017) highlighting “mask anxiety” as a prevalent clinical concern, particularly during initial treatment sessions [5,6]. In addition to psychological stress, certain medical scenarios necessitate mask modifications. Patients requiring nasogastric or tracheostomy tubes, oral bite blocks, or those with facial wounds or sensitive skin often need custom cutouts to ensure safety and comfort [7]. However, such modifications can compromise the structural integrity of the mask, potentially reducing its immobilization effectiveness, a trade-off that must be carefully managed [8,9]. Moreover, the fabrication process itself, which typically lasts 30–60 min, further adds to patient burden, especially given the need for immobility during molding [4]. Although thermoplastic masks generally offer reliable daily reproducibility, minor inter-fraction variations are common [10]. These deviations, often in the range of a few millimeters, can arise not only from patient repositioning but also from fabrication-related uncertainties such as inconsistent heating, stretching, and cooling during molding. Such geometric inaccuracies necessitate additional safety margins around the tumor, which can increase radiation exposure to adjacent healthy tissues [11].

Digital design workflows and additive manufacturing have been proposed as promising approaches to address several limitations of conventional immobilization masks [12,13,14,15]. Using patient imaging data (e.g., CT or MRI), immobilization devices can be digitally designed prior to fabrication, enabling customization of geometry, ventilation patterns, and facial openings [16,17]. Such workflows offer the potential to reduce patient-present molding, improve comfort, and enhance design flexibility. Acrylonitrile butadiene styrene (ABS) and its composites have emerged as strong candidates for use in radiotherapy immobilization masks due to their combination of mechanical robustness and radiological compatibility [1,18]. A previous study by Jreije et al. (2024) found that ABS-based composites exhibit mechanical properties comparable to those of commercial thermoplastics while providing enhanced resistance to ionizing radiation within clinically relevant dose ranges [18]. In particular, bismuth oxide (Bi_2_O_3_)-filled ABS has demonstrated minimal degradation in structural integrity following exposure to therapeutic photon beams, making it suitable for repeated clinical use. These advantages underscore the potential of ABS-based composites as viable alternatives to traditional thermoplastic materials in the development of next-generation immobilization devices for precision radiotherapy.

This study aimed to mechanically evaluate customizable head and neck immobilization mask designs based on ABS and ABS/Bi_2_O_3_ composites and to compare their performance with commercial thermoplastic masks. The novelty of this study lies in combining: (i) a systematic experimental comparison of the mechanical properties of ABS and Bi_2_O_3_-reinforced composites against clinically used thermoplastic masks, including an assessment of material stability after multiple recycling cycles; (ii) the development of a fully customizable, ergonomically optimized digital mask design with variable thickness and reinforced structural regions; and (iii) comprehensive finite element simulations using clinically realistic neck-force loading conditions to quantify stress distribution and displacement behavior. While additive manufacturing is discussed as a clinically relevant fabrication pathway, the present work focuses on mechanical modeling and comparative performance assessment, providing a foundation for future manufacturing-calibrated and clinically validated implementations.

## 2. Materials and Methods

### 2.1. Production of 3D Printing Filaments

ABS composite filaments containing 5 wt%, 10 wt%, and 20 wt% bismuth oxide (Bi_2_O_3_) additives were produced following the methodology described in our previous work [18]. Initially, ABS pellets (3Devo Filament Maker, 3Devo, Utrecht, The Netherlands) and Bi_2_O_3_ powder (Bi_2_O_3_, <200 nm, 99.999% trace metals basis, Merck, Germany) were dry-mixed and extruded using a single-screw extruder (Precision 350, 3Devo Filament Maker, Utrecht, The Netherlands) to produce preliminary filament. To enhance the homogeneity of the composites, the extruded filament was subsequently shredded and ground into flakes using a SHR3D IT shredder (3Devo, Utrecht, The Netherlands). These regrinds were dried at 50 °C for several hours to eliminate residual moisture.

A second extrusion process was then performed using the same extruder to produce final filaments with a diameter of 1.75 ± 0.1 mm. The extrusion temperature profile for ABS composites was chosen as follows: preheating at 240 °C, melting at 230 °C, shear zone at 220 °C, and extrusion at 215 °C. Filament spooling was performed after the extruded filament reached dimensional and thermal stability. Virgin ABS filaments were prepared using the same protocol for direct comparison.

### 2.2. Mechanical Testing of Materials

The mechanical properties of six different materials were evaluated: four in-house-produced 3D printing filaments (ABS and ABS/Bi_2_O_3_ 5 wt%, 10 wt%, and 20 wt%) and two commercially available thermoplastic immobilization mask materials. For each material, three sets of standardized tensile test specimens were prepared and tested.

Tensile test specimens from each 3D printing material were printed according to the ISO 527-2:2025 standard (Type 1A dog bone geometry) [19]. A fused deposition modeling 3D printer, Zortrax M300 (Zortrax, Olsztyn, Poland), was used for all sample production. Printing parameters were set as follows: nozzle temperature of 230–270 °C, bed temperature of 80 °C, 100% solid infill, and 0.29 mm layer height. These conditions were optimized to minimize print defects and ensure mechanical integrity.

The commercial thermoplastic masks assessed in this study were Orfit 35768/2MI/FOAM (Orfit Industries, Wijnegem, Belgium; referred to as CTM1 thereafter), a 3-point head immobilization mask of 2 mm thickness constructed from a micro-perforated thermoplastic material, and Macromedics MacroCast Fine Perfo (REMCF-3) (Macromedics Holding B.V., Moordrecht, The Netherlands; referred to as CTM2 thereafter), a pre-cut, 3-point head mask design with a similar 2 mm thickness and micro-perforated structure. Both mask models were fixed to the treatment table using pushpins.

**Figure 1:**
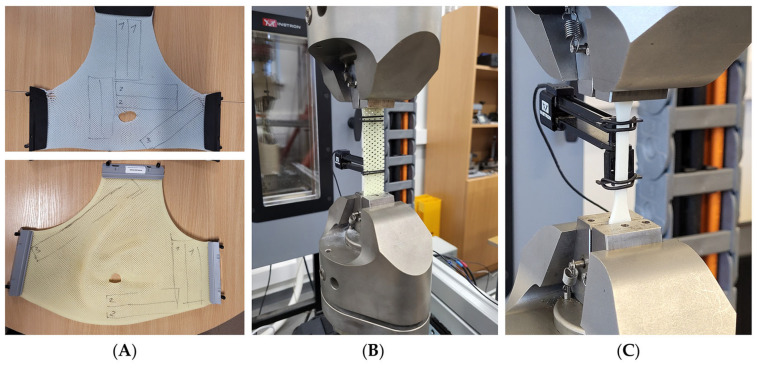
MacroMedics^®^ (**above**) and Orfit^®^ (**below**) head immobilization masks used for tensile tests with the indicated sampling areas (**A**); Instron ElectroPuls^®^ Linear–Torsion machine with an inserted sample from commercial mask (**B**) and 3D printed sample (**C**).

In order to perform tensile tests with the mask materials, rectangular/flat specimens were cut from the masks with dimensions conforming to a gauge length of 80 mm, overall length of 150 mm, and a cross-sectional area of 20 mm × 2 mm (Figure 1). Samples were extracted from various mask regions and orientations (0°, 45°, and 90° relative to the mask’s manufacturing direction) to account for potential anisotropy. Both commercial masks had previously been used in full clinical treatment cycles for H&N cancer radiotherapy involving 12 treatment fractions. As a result, they had been subjected to activation in hot water, stretching, and conformal molding on patients, mimicking typical clinical usage conditions. It is important to note that manufacturers disclose neither the exact chemical composition nor mechanical specifications of their thermoplastic materials. Therefore, mechanical testing was performed to determine baseline properties, which could serve as reference data for the evaluation of 3D-printed mask alternatives.

Tensile tests were conducted using an ElectroPuls^®^ E10000 Linear–Torsion testing machine (Instron, Norwood, MA, USA), following the procedures outlined in ISO 527-1:2019 [20]. Axial strain was measured using a clip-on extensometer (Instron 2620, class 0.5 accuracy) with a gauge length of 50 mm. For each material type, three tensile specimens (*n* = 3) were tested. The mechanical parameters relevant to this study, including yield strength and Young’s modulus, were obtained, and results are reported as mean values with corresponding standard deviations. If the standard deviation exceeded 15% of the mean, additional specimens were tested to ensure consistency.

### 2.3. Recycling of 3D Printing Materials

To assess the reusability and mechanical stability of ABS-based composites under repeated manufacturing cycles, recycling tests were performed on virgin ABS and Bi_2_O_3_-filled composites (5 wt% and 10 wt%). Printed specimens were mechanically granulated and reprocessed through multiple extrusion–printing cycles, simulating repeated reuse of material in a closed-loop production workflow. Each cycle involved mechanical grinding of previously printed specimens into small flakes (≈3–5 mm) using 3Devo SHR3D IT (Utrecht, The Netherlands), followed by thermal re-extrusion of the shredded material into new 1.75 mm filaments and then reprinting of new tensile specimens using an FDM 3D printer under identical process parameters as for the virgin samples. This procedure was repeated for five and ten consecutive cycles for each material formulation to represent moderate and extended reprocessing conditions, respectively.

The resulting filaments and printed samples were visually inspected for surface homogeneity and color change, then conditioned at 24 °C and 50% relative humidity for 24 h before mechanical testing. Tensile tests were subsequently performed according to the same protocol described in Section 2.2 (Mechanical Testing of Materials), using three specimens per condition to obtain mean and standard deviation values for Young’s modulus and yield stress.

### 2.4. Development of a Virtual Head Immobilization Mask

**Figure 2:**
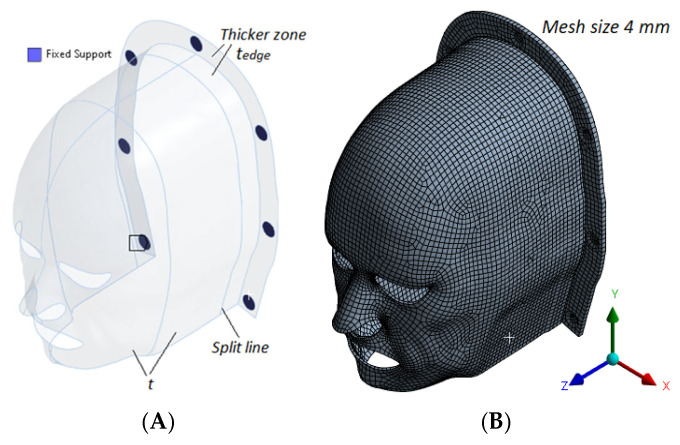
Patient-specific mask model. (**A**) Digital mask design showing the nominal thickness (*t*), reinforced edge thickness (*t_edge_*), split line, and fixed support regions. (**B**) Finite element mesh of the head–mask assembly with a uniform mesh size of 4 mm.

A virtual patient-specific head immobilization mask was designed using a digital human model representative of an average middle-aged male. The anatomical model was generated using MakeHuman 1.3.0 (an open-source software for creating 3D human meshes) [21] and imported into the open-source 3D computer graphics software Blender 4.2 for further editing [22]. The head geometry was sectioned along a coronal plane near the ears to isolate the anterior face region. To improve comfort and reduce the feeling of claustrophobia, the model was adapted by introducing cutouts at the eye, nasal, and oral regions. Additionally, a 2 mm uniform offset was applied for an optimal mask–patient skin fit. A U-shaped support base was integrated into the mask design with fixation points for attachment to the treatment table. The final model was exported as a high-resolution polygonal mesh, consistent with the resolution required for computational analysis and 3D printing (Figure 2).

### 2.5. Finite Element Analysis of Mechanical Performance

**Figure 3:**
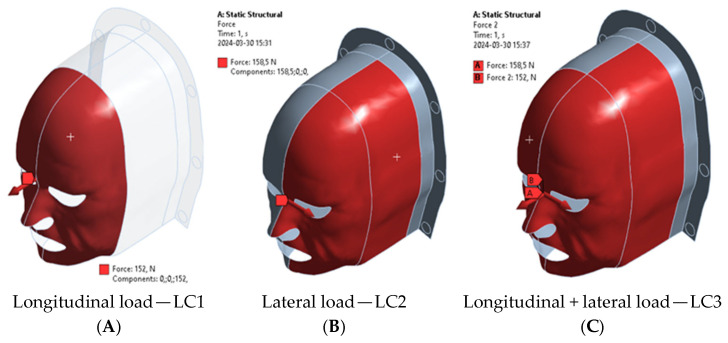
Illustration of the load under neck flexion (**A**), left lateral bending of the neck (**B**), and both neck movements (**C**).

The static mechanical behavior of the designed immobilization masks was evaluated using Finite Element Analysis (FEA) in ANSYS Workbench (Ansys Inc., Canonsburg, PA, USA). The objective was to assess the stress distribution and displacement response of the masks under patient-induced loading scenarios. Three primary loading cases (LC) were simulated based on the force values reported by Almosnino et al. 2010 [23], who characterized neck muscle exertion under isometric conditions: Lateral neck bending (LC1)—158.5 N applied to the side of the mask; Neck flexion (LC2)—152 N applied to the anterior region of the mask; Combined lateral and forward flexion (LC3)—a resultant vector load of $\sqrt{158.5^{2}+152^{2}}$ N (Figure 3).

Boundary conditions were applied to the mask’s fixation zones, representing the immobilization pins connecting the mask to the treatment couch. All degrees of freedom were constrained in these regions to simulate a rigid fixation scenario. The analysis was performed for masks of three different thicknesses: 1 mm, 2 mm, and 4 mm, to examine the effect of thickness on mechanical performance. The materials simulated in the model included commercial thermoplastics (based on experimentally derived values from Orfit^®^ and Macromedics^®^ masks) and in-house-developed 3D printing composites (ABS and ABS/Bi_2_O_3_ at 5%, 10%, and 20% weight fractions).

Each material was modeled as linear elastic, with input parameters such as Young’s modulus, Poisson ratio and yield stress obtained from experimental tensile tests. The primary mechanical performance indicators evaluated in the FEA were equivalent (von Mises) stress, used to verify that stress levels remained below yield limits, and resultant displacement, which was used to quantify how much the mask deformed under load, directly impacting its immobilization effectiveness.

To assess the effectiveness of the material distribution, the mask model was divided into two zones of different thickness. This approach allowed evaluation of locally reinforced designs, such as thicker mask edges for enhanced fixation near attachment points.

### 2.6. Statistical Analysis

To evaluate whether yield strength significantly differed among materials, a one-way analysis of variance (ANOVA) followed by Tukey’s Honestly Significant Difference (HSD) post hoc test was conducted.

All statistical analyses were conducted using R software (version 4.3.1), with statistical significance defined as *p* < 0.05.

## 3. Results and Discussion

**Figure 4:**
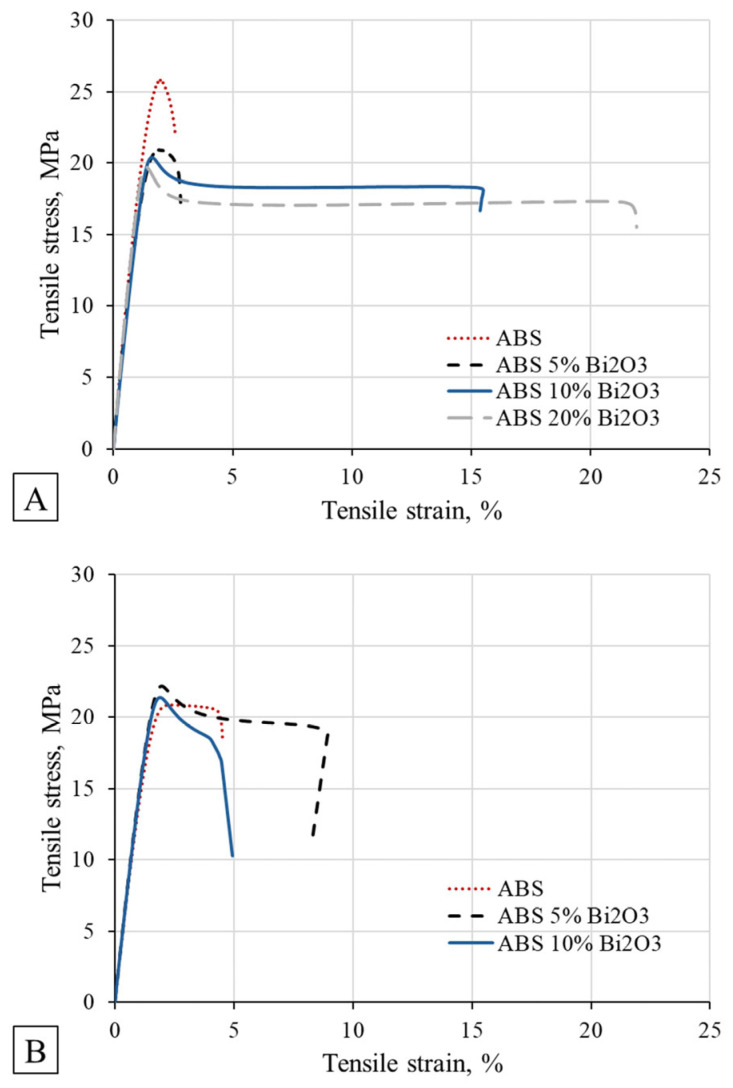
Tensile stress vs. strain graphs of 3D printing composites before (**A**) and after recycling 10 times (**B**).

The mechanical properties of six materials, including four custom-developed ABS-based composites and two commercial thermoplastic masks, were evaluated (Table 1). The ABS-based materials reinforced with 5%, 10%, and 20% Bi_2_O_3_ displayed a range of mechanical stiffness and yield strengths. The stress–strain curves of ABS-based composites are shown in Figure 4. Virgin ABS exhibited the highest Young’s modulus (2.5 GPa), while commercial thermoplastic masks showed significantly lower stiffness values (0.25–0.3 GPa). The incorporation of Bi_2_O_3_ slightly reduced the stiffness compared to virgin ABS, but all composites maintained a Moduli above 1.7 GPa, indicating that structural rigidity was largely preserved.

In terms of yield strength, the ABS-based materials again outperformed commercial alternatives. Virgin ABS exhibited a yield strength of approximately 25 MPa, while composites with 5–20 wt% Bi_2_O_3_ exhibited values around 20 MPa. This suggests that the addition of bismuth filler did not significantly compromise the material’s capacity to withstand permanent deformation. In contrast, commercial masks showed substantially lower yield strengths at approximately 7 MPa, consistent with their lower stiffness. These differences were statistically significant, as confirmed by One-Way ANOVA (*p* < 0.001). Further pairwise comparison using Tukey’s HSD post hoc test revealed that ABS exhibited significantly higher yield strength than all other materials (*p* < 0.001). These findings suggest that the thermoplastic materials used in conventional masks may deform under modest patient-induced forces, whereas ABS-based materials have superior load-bearing capacity and structural resilience.

These findings are consistent with earlier reports that traditional thermoplastic masks, while adequate for clinical use, are often limited in strength and rigidity [4,8]. These masks are typically made from low-melting-point polymers that become pliable when heated and rely on the material’s residual shrinkage and surface adhesion to conform tightly to the patient’s anatomy [24]. Importantly, the commercial masks tested in our study had already been used throughout full clinical treatment courses involving 12 fractions, meaning they had undergone the standard sequence of thermal activation, stretching, and patient-specific molding. This reflects realistic clinical usage conditions and aligns with observations by Haefner et al. (2018), who reported that repeated thermal activations during routine mask preparation and adjustment can reduce stiffness and shape fidelity over time, potentially compromising immobilization accuracy during multi-fraction treatments [1]. In contrast, the ABS composites used in this study exhibited stable, reproducible mechanical behavior and offered a durable alternative that can better withstand long-term clinical use without structural fatigue.

To evaluate material sustainability and mechanical stability during repeated material recycling, the mechanical performance of recycled ABS and ABS/Bi_2_O_3_ composites was assessed after multiple extrusion–printing cycles. The results are summarized in Table 2. For virgin ABS, recycling led to a gradual reduction in stiffness and yield strength. Young’s modulus decreased from 2.50 ± 0.13 GPa to 1.57 ± 0.15 GPa after five cycles and 1.41 ± 0.11 GPa after ten cycles, while yield stress declined from 25.1 ± 1.9 MPa to 22.9 ± 1.7 MPa and 20.9 ± 1.2 MPa, respectively. This reduction is attributed to polymer chain scission and minor thermal oxidation during reprocessing, phenomena commonly observed in amorphous thermoplastics such as ABS [25].

In contrast, ABS/Bi_2_O_3_ composites displayed greater mechanical stability during recycling. The addition of bismuth oxide reduced the rate of stiffness loss, and, notably, yield strength values remained nearly constant, or even slightly improved, after multiple processing cycles. For example, ABS/5% Bi_2_O_3_ exhibited a Young’s modulus of 1.83 ± 0.10 GPa and a yield stress of 25.4 ± 1.4 MPa after five cycles, maintaining mechanical performance comparable to or better than the virgin composite. Even after ten cycles, both ABS/5% Bi_2_O_3_ and ABS/10% Bi_2_O_3_ retained moduli above 1.5 GPa and yield strengths of approximately 22 MPa, exceeding those of recycled neat ABS.

These results indicate that Bi_2_O_3_ reinforcement enhances the thermal and mechanical stability of ABS during repeated processing, likely by mitigating molecular degradation and improving filler–matrix heat dissipation [26,27]. Consequently, the composites can be reprocessed multiple times without losing their suitability for use in future immobilization mask fabrication. This recyclability supports the development of closed-loop, resource-efficient manufacturing workflows and aligns with circular-economy principles for sustainable medical device production.

**Figure 5:**
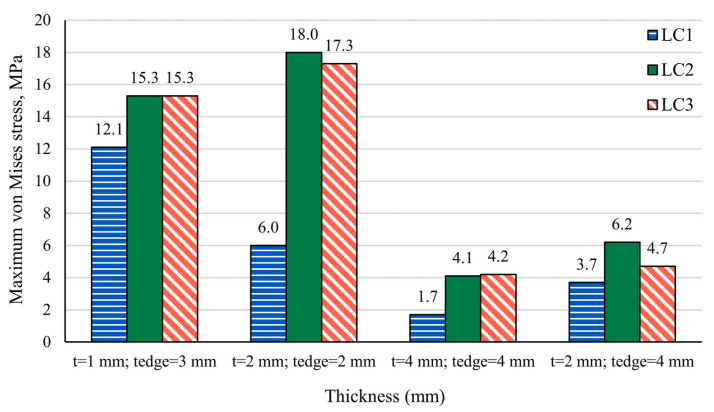
Maximum von Mises stress for 2 mm and 4 mm thick immobilization masks following Longitudinal loading—LC1, Lateral loading—LC2, and Combined longitudinal and lateral loading—LC3.

**Figure 6:**
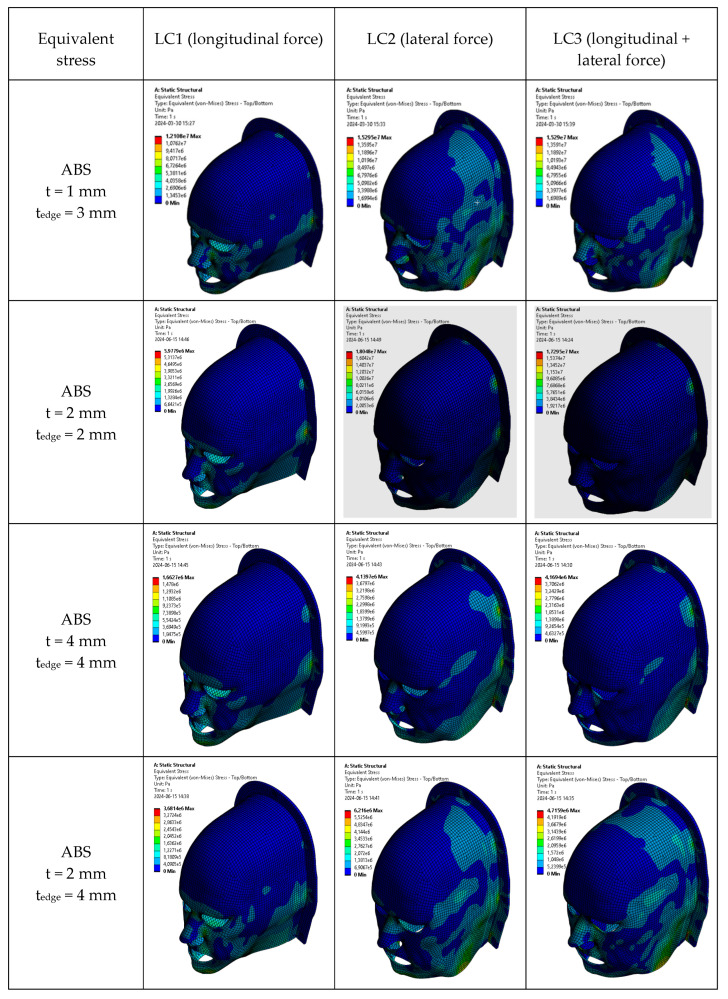
Modeling results for maximum von Mises stress (equivalent stress) in ABS-based immobilization masks of different thicknesses.

Finite Element Analysis (FEA) simulations were conducted to evaluate maximum von Mises stress under three loading scenarios: longitudinal (LC1), lateral (LC2), and combined (LC3) forces. Four different scenarios involving various immobilization mask thicknesses and edge thicknesses were simulated. In the combined loading case (LC3), the applied force components acted in different directions, resulting in partial cancellation of deformation vectors and, consequently, reduced resultant displacement and equivalent stress compared to the corresponding single-load cases. Results are summarized in Figure 5 and visualized in Figure 6.

Across all loading scenarios, ABS-based masks remained below their yield strength even at the thinnest configuration (t = 1 mm), confirming that these materials operate safely within the elastic regime. Increasing the mask thickness (t = 2 mm or t = 4 mm) further reduced stress values for all load cases. For the hybrid configuration (t = 2 mm, t_edge_ = 4 mm), stresses also decreased relative to the uniform 2 mm mask, reflecting the contribution of the thicker edges in redistributing load. As with the other ABS-based configurations, the hybrid model remained well below yield strength for all loading scenarios.

Stress distributions for home-extruded ABS are shown in Figure 6. Differences in mask-body versus edge thickness produced slightly less homogeneous stress fields, but this effect was geometric rather than material-dependent. As expected and consistent with our tensile characterization, varying the concentration of Bi_2_O_3_ did not noticeably influence the maximum von Mises stress values, since stress magnitudes in FEA are governed primarily by load magnitude and geometry rather than modest variations in stiffness.

In contrast, for the commercial thermoplastic materials, the same load and geometric conditions produced stresses exceeding the yield strength at thinner geometries. Specifically, for a thickness of 1 mm, the commercial material exceeded its yield strength in all three loading cases (LC1–LC3), and for 2 mm thickness, the stresses under lateral (LC2) and combined (LC3) loading were still above the yield limit. Only at 4 mm thickness did the maximum stresses fall below the yield strength in all load cases, although they remained closer to the yield point than in the ABS-based configurations. These results indicate that, under conservative neck-load conditions, commercial thermoplastic masks are more prone to entering the plastic regime at clinically relevant thin geometries, whereas ABS-based masks remain in the elastic domain over the same range of thicknesses.

**Figure 7:**
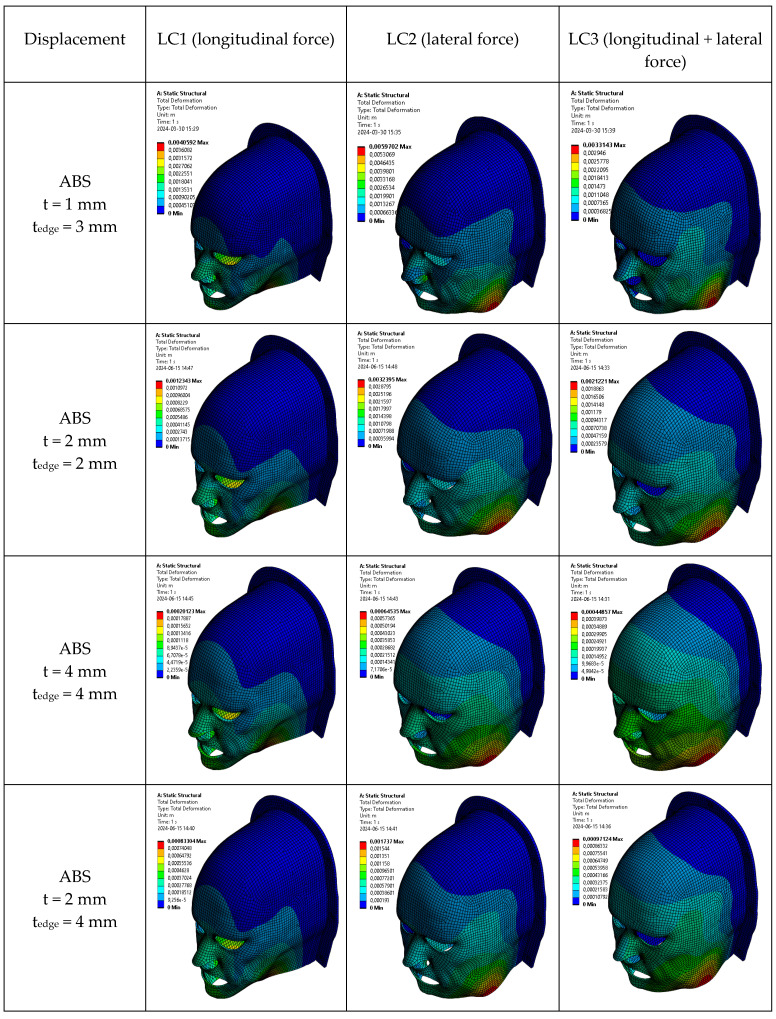
Modeling results for resulting displacement in ABS-made masks under stress.

Figure 7 and Table 3 report the maximum displacement observed under the same loading scenarios. For the thinnest mask configuration (1 mm), displacement values were highest across all ABS materials, especially under lateral loading, where the geometry provides the least resistance. In contrast, at 2 mm thickness, displacement was drastically reduced. ABS and its composites exhibited displacements between 1.2 mm and 4.8 mm, whereas commercial masks showed significantly higher displacement, reaching values above 25 mm under lateral load, which is well beyond clinically acceptable immobilization margins.

At 4 mm, all materials demonstrated excellent mechanical performance, with displacements in ABS-based masks dropping to as low as 0.20 mm under longitudinal loading and 0.6–1.0 mm under lateral loading. These values fall within or below the sub-millimeter precision required in advanced radiotherapy techniques such as stereotactic radiotherapy. Importantly, even with the hybrid configuration (2 mm thick body with 4 mm reinforced edges), ABS-based designs maintained displacement values around 1–2 mm, offering a balance between rigidity and material efficiency.

These results echo prior findings by Fisher et al. (2020), who demonstrated that a 4 mm thick 3D-printed mask developed from CT data could maintain head positioning within ±4 mm on a phantom [28]. More recently, Chen et al. (2023) developed custom 3D-printed masks integrated with bite blocks and headrests and showed sub-millimeter reproducibility during stereotactic treatment [29]. While these studies demonstrated acceptable static immobilization, they did not assess dynamic stress or displacement under load. Our study fills this gap by simulating real-time forces that may be exerted by patients (e.g., via muscle contraction or discomfort-driven movement), providing a more comprehensive mechanical validation.

The displacement results across the ABS/Bi_2_O_3_ composites do not follow a monotonic trend with increasing filler percentage. This behavior is explained by the measured Young’s moduli of the materials: the 5% Bi_2_O_3_ composite exhibited a slightly higher modulus than the 10% formulation, resulting in correspondingly lower displacement in the FEA. Because maximum displacement in linear elastic analysis is inversely proportional to stiffness, small modulus variations between formulations lead to minor non-systematic differences. These variations fall within expected experimental scatter and do not indicate any detrimental mechanical effect from increasing Bi_2_O_3_ content. Importantly, all ABS-based materials, regardless of filler content, performed substantially better than commercial thermoplastics.

Furthermore, although pure ABS demonstrated the lowest displacement overall, the ABS/Bi_2_O_3_ composites remain equally viable alternatives, offering comparable displacement performance while also providing enhanced recyclability due to their improved thermal stability during repeated extrusion cycles.

The mechanical performance improved markedly with increasing mask thickness. However, overly rigid masks can reduce patient comfort and may not conform well to anatomical irregularities. Therefore, the hybrid configuration featuring a 2 mm thick body with 4 mm thick edge reinforcements was investigated in this work. The mask edge is part of the mask that merges with the U-shaped fixture dedicated to fixing the mask to the table. This configuration also demonstrated promising results: ABS and its composites exhibited reduced displacement (around 1–2 mm under LC1/LC2), thus providing structural stability while optimizing material usage and printing time.

As can be seen from Figure 7, the maximum displacement occurs in the lower jaw region across all the loading cases. This localized phenomenon is due to the larger free edge in this area. Moreover, other regions of the mask exhibit stiffer behavior, especially the forehead. To improve the stiffness in the lower jaw region, local thickening of the mask could be performed while keeping in mind the effect of any mask modification on radiation attenuation. In particular, for all masks, the maximum displacement at the load case (LC3) when both orthogonal loads are applied simultaneously is smaller compared to the load cases when loads are applied separately. When a force is applied in a single direction (LC1 or LC2), the mask undergoes not only displacement in the force direction but also a pronounced secondary deformation in the perpendicular direction, due to bending and ovalization of the thin-wall structure. This is analogous to a ring that flattens laterally when compressed vertically. When orthogonal loads are applied simultaneously, these deformation modes interact and partially constrain one another, increasing effective geometric stiffness and resulting in a reduced maximum resultant displacement compared with the single-load cases, despite the higher overall force. This effect reflects geometric coupling rather than force cancellation and is characteristic of ring-like and thin-shell structures under multi-axial loading.

The improved mechanical performance of ABS-based 3D-printed masks has several clinical implications. First, enhanced rigidity reduces the need for large planning margins, potentially lowering radiation exposure to adjacent healthy tissues. Second, the digital design workflow eliminates the need for patient-present molding, reducing anxiety and discomfort. Ventilation cutouts and ergonomic customization may further enhance patient tolerance compared with closed thermoplastic masks. However, excessive rigidity could compromise comfort if the mask does not adequately conform to anatomical irregularities. Our results suggest that hybrid reinforcement—such as thicker edges combined with thinner central areas—can balance immobilization stability with adaptability. Moreover, localized reinforcement of the jaw region, which showed the greatest displacement, may improve performance without sacrificing comfort.

While this study provides robust simulation and material testing data, several limitations must be acknowledged. The simulations assumed linear elastic behavior and ideal attachment to the treatment table, without modeling skin–mask interactions or real-time motion. Additionally, the influence of repeated thermal cycling and cleaning on long-term mechanical integrity remains to be studied. Another limitation is that the mechanical properties were obtained from tensile specimens printed in a flat orientation; however, a full immobilization mask cannot be printed entirely in this orientation due to its complex curved geometry. As a result, real printed masks may exhibit local anisotropies that are not captured in the material characterization or in the FEA, which assumes homogeneous material behavior. Future work should incorporate experimental motion tracking (e.g., with surface-guided radiation therapy (SGRT) or cone-beam computed tomography (CBCT)) on patients or anthropomorphic phantoms wearing the 3D-printed masks to validate clinical reproducibility under real-world conditions. Moreover, while the head model used in simulation represents a generic average male anatomy, anatomical variability in clinical populations (e.g., pediatric patients, those with facial deformities, or postoperative swelling) may affect the generalizability of the results. Follow-up studies should investigate mask performance on a broader range of patient-specific geometries.

Finally, while this study focused on mechanical properties, future work should address workflow integration, cost–benefit analyses, and radiation dosimetry to fully establish the clinical viability of 3D-printed immobilization masks. Experimental motion tracking using surface-guided radiotherapy (SGRT) or cone-beam CT should be performed to validate immobilization accuracy in clinical or phantom trials. Long-term testing of material behavior under repeated sterilization and irradiation cycles will also be critical.

## 4. Conclusions

This study demonstrated that patient-specific immobilization masks fabricated from ABS-based composites provide a substantial improvement in mechanical performance compared with conventional thermoplastic masks used in head and neck radiotherapy. Tensile testing confirmed that ABS and Bi_2_O_3_-reinforced composites achieved significantly higher stiffness (1.7–2.5 GPa) and yield strength (20–25 MPa) relative to commercial thermoplastics (0.25–0.3 GPa and ~7 MPa, respectively). In addition, recycling tests revealed that while virgin ABS exhibited moderate decreases in stiffness and strength after multiple reprocessing cycles, Bi_2_O_3_-filled composites maintained their yield strength and showed only limited reductions in stiffness even after ten extrusion–printing cycles. These results demonstrate superior structural resilience and reusability of ABS composites and a markedly reduced likelihood of permanent deformation under clinically relevant stresses.

Finite element simulations further highlighted the advantages of ABS-based masks. At 2 mm thickness, displacements remained within 1–5 mm, while 4 mm masks achieved sub-millimeter stability, surpassing the precision thresholds required for advanced radiotherapy. In contrast, conventional thermoplastic masks displayed displacements exceeding 10 mm under longitudinal force and 25 mm under lateral loading, often approaching or surpassing their material yield points. Importantly, hybrid designs with thinner central regions and locally reinforced 4 mm edges effectively reduced displacement to 1–2 mm, while maintaining ergonomic adaptability and optimizing material usage.

Together, these results identify pure ABS as the baseline material for the final mask design due to its slightly superior displacement performance. However, ABS/Bi_2_O_3_ composites remain strong and practical alternatives, offering comparable mechanical performance while providing improved recyclability and enhanced stability during repeated reprocessing—features that align with sustainable manufacturing and circular-economy goals.

These findings underscore the clinical potential of ABS-based masks to reduce planning target volume margins, thereby limiting unnecessary radiation exposure to organs at risk. While further validation in clinical and phantom-based motion tracking studies is necessary, alongside long-term durability and dosimetric evaluations, this work establishes ABS-based composites as promising candidates for next-generation immobilization devices in precision radiotherapy. Their integration into clinical workflows has the potential to enhance treatment accuracy, patient experience, and sustainability in medical device manufacturing.

## Figures and Tables

**Table 1:** Properties of materials used in mask simulations. One-way ANOVA: F(5,12) = 9.54 × 10^30^, *p* < 0.0001.

Properties	Material					
	CTM1	CTM2	ABS	ABS/5% Bi_2_O_3_	ABS/10% Bi_2_O_3_	ABS/20% Bi_2_O_3_
Yield stress, σ_y_ (MPa)	7 ± 0.6 ^a^	7 ± 0.5 ^a^	25 ± 1.9 ^b^	21 ± 1.7 ^c^	20 ± 2.1 ^cd^	20 ± 2.2 ^cd^
Young’s modulus, YM (GPa)	0.25 ± 0.02	0.30 ± 0.02	2.5 ± 0.1	1.9 ± 0.1	1.70 ± 0.09	2.0 ± 0.1

^a–d^ denote results of the post hoc statistical analysis; values with identical letters show no significant difference (*p* < 0.05).

**Table 2:** Retention of mechanical properties of ABS and ABS/Bi_2_O_3_ composites after repeated recycling cycles (values relative to virgin material).

Material	Cycles	Young’s Modulus (GPa)	% of Virgin	Yield Stress (MPa)	% of Virgin
ABS	5×	1.57 ± 0.15	63%	22.9 ± 1.7	91%
	10×	1.41 ± 0.11	56%	20.9 ± 1.2	83%
ABS/5% Bi_2_O_3_	5×	1.83 ± 0.10	97%	25.4 ± 1.4	121%
	10×	1.55 ± 0.09	82%	22.2 ± 1.7	106%
ABS/10% Bi_2_O_3_	5×	1.50 ± 0.09	86%	20.0 ± 1.9	98%
	10×	1.53 ± 0.14	88%	21.4 ± 2.1	105%

**Table 3:** Maximum resulting displacement (mm) for 1 mm, 2 mm, and 4 mm thick immobilization masks. LC1 = Longitudinal loading; LC2 = Lateral loading; LC3 = Combined longitudinal and lateral loading.

Mask’s Thickness (mm)	Material	Displacement, mm		
		LC1	LC2	LC3
t = 1 mm t_edge_ = 3 mm	ABS	4.06	5.97	3.31
	ABS/5% Bi_2_O_3_	5.34	7.86	4.36
	ABS/10% Bi_2_O_3_	5.97	8.78	4.87
	ABS/20% Bi_2_O_3_	5.08	7.46	4.14
t = 2 mm t_edge_ = 2 mm	CTM1	12.30	32.40	21.20
	CTM2	10.25	27.00	17.67
	ABS	1.23	3.24	2.12
	ABS/5% Bi_2_O_3_	1.62	4.26	2.79
	ABS/10% Bi_2_O_3_	1.81	4.76	3.12
	ABS/20% Bi_2_O_3_	1.54	4.05	2.65
t = 4 mm t_edge_ = 4 mm	CTM1	2.01	6.45	4.49
	CTM2	1.67	5.38-	3.74
	ABS	0.20	0.65	0.45
	ABS/5% Bi_2_O_3_	0.26	0.85	0.59
	ABS/10% Bi_2_O_3_	0.29	0.95	0.66
	ABS/20% Bi_2_O_3_	0.25	0.81	0.56
t = 2 mm t_edge_ = 4 mm	ABS	0.83	1.74	0.97
	ABS/5% Bi_2_O_3_	1.09	2.29	1.28
	ABS/10% Bi_2_O_3_	1.22	2.56	1.43
	ABS/20% Bi_2_O_3_	1.04	2.18	1.21

## Data Availability

The original contributions presented in the study are included in the article. Further inquiries can be directed to the corresponding authors.

## Abbreviations

The following abbreviations are used in this manuscript:

H&N	Head and neck
CT	Computed Tomography
MRI	Magnetic resonance imaging
ABS	Acrylonitrile butadiene styrene
Bi_2_O_3_	Bismuth oxide
FEA	Finite element analysis
